# Modeling the costs and benefits of temporary recommendations for poliovirus exporting countries to vaccinate international travelers

**DOI:** 10.1016/j.vaccine.2017.05.090

**Published:** 2017-07-05

**Authors:** Radboud J. Duintjer Tebbens, Kimberly M. Thompson

**Affiliations:** Kid Risk, Inc., Orlando, FL, USA

**Keywords:** Traveler vaccination, Health economics, Polio eradication, Outbreaks, International Health Regulations, cMYP, comprehensive multi-year plan, cVDPV(2), circulating vaccine-derived poliovirus (of serotype 2), HF, health facility, IHREC, International Health Regulations Emergency Committee, INB, incremental net benefit, IPV, inactivated poliovirus vaccine, POE, point of entry, OPV, oral poliovirus vaccine, oSIA, outbreak response supplemental immunization activity, pSIA, planned preventive supplemental immunization activity, TR, temporary recommendations, WHO, World Health Organization, WPV1, serotype 1 wild poliovirus, $, year 2015 United States dollars

## Abstract

Recognizing that infectious agents readily cross international borders, the International Health Regulations Emergency Committee issues Temporary Recommendations (TRs) that include vaccination of travelers from countries affected by public health emergencies, including serotype 1 wild polioviruses (WPV1s). This analysis estimates the costs and benefits of TRs implemented by countries with reported WPV1 during 2014–2016 while accounting for numerous uncertainties. We estimate the TR costs based on programmatic data and prior economic analyses and TR benefits by simulating potential WPV1 outbreaks in the absence of the TRs using the rate and extent of WPV1 importation outbreaks per reported WPV1 case during 2004–2013 and the number of reported WPV1 cases that occurred in countries with active TRs. The benefits of TRs outweigh the costs in 77% of model iterations, resulting in expected incremental net economic benefits of $210 million. Inclusion of indirect costs increases the costs by 13%, the expected savings from prevented outbreaks by 4%, and the expected incremental net benefits by 3%. Despite the considerable costs of implementing TRs, this study provides health and economic justification for these investments in the context of managing a disease in advanced stages of its global eradication.

## 1 Introduction

Recognizing that infectious agents readily cross international borders, the World Health Organization (WHO) International Health Regulations Emergency Committee (IHREC) issues Temporary Recommendations (TRs), which include requirements to vaccinate travelers from countries affected by public health emergencies. Between May 2014 and the end of 2016, the IHREC for polio issued TRs to five countries experiencing WPV1 transmission (i.e., Afghanistan, Cameroon, Equatorial Guinea, Pakistan, and the Syrian Arab Republic) [1], [2]. Of these, only Pakistan, Afghanistan, and Cameroon provided evidence to the WHO of substantive implementation of the TRs, with Pakistan demonstrating the most extensive efforts. To date, no known new WPV1 outbreaks occurred as a result of WPV1 exportations from these countries, although cross-border transmission between Pakistan and Afghanistan continued to occur on a background of ongoing indigenous WPV1 transmission in both countries. In contrast, outbreaks associated with WPV1 importations regularly occurred in previously polio-free countries in the 10-year period preceding the first polio TRs [3]. This could reflect the reduced overall incidence of WPV1 (possibly in part motivated by the TRs), improvement by polio-free countries to manage their population immunity to serotype 1 poliovirus transmission, and/or effectiveness of the TRs in reducing WPV1 exportation risks. The TRs may reduce WPV1 exportations by immunizing previously unvaccinated travelers or boosting the immunity of travelers with waned immunity, both of which reduce the probability and duration of any WPV1 infections they may acquire before traveling to another country [4].

Few studies estimate the costs and benefits of traveler recommendations for infectious diseases [5], [6], and no prior published studies explore the economics of TRs for polio, although some assessed the risk of international poliovirus spread [7], [8]. The WHO compiled unpublished data that estimated TR vaccination costs of approximately $1.5 million per year including vaccine and personnel costs at points of entry (POEs), but only vaccine costs for traveler vaccinations administered at health facilities (HFs). WHO data further suggest costs to respond to outbreaks in previously polio-free countries of $850 million during 2003–2009 [8] and $1.15 billion during 2003–2014 [9]. Recognizing that countries need to budget for the costs of implementing TRs, but not for the unobservable benefits of prevented outbreaks and cases, questions remain about how the costs of the TRs compare to their health and economic benefits. This analysis used a decision analytic model to estimate the economic trade-offs associated with implementation of the recent polio TRs.

## 2 Methods

We focus on the costs and benefits of the TRs during the 3 years 2014–2016 because the IHREC for polio issued the first TRs for polio in May 2014. We consider the possibility of prevented outbreaks (defined as one or more reported polio cases linked to a WPV1 importation into a previously polio-free country and excluding circulating vaccine-derived poliovirus (cVDPV) outbreaks) for up to 10 years (i.e., through the end of 2023) and the expected lifetime societal benefits of prevented polio cases. We report all monetary outcomes in year 2015 US dollars ($) and discount using a rate of 3% [10] from the perspective of a decision maker in 2014. We include all costs regardless of who pays for them (e.g., country, Global Polio Eradication Initiative).

**Fig. 1:**
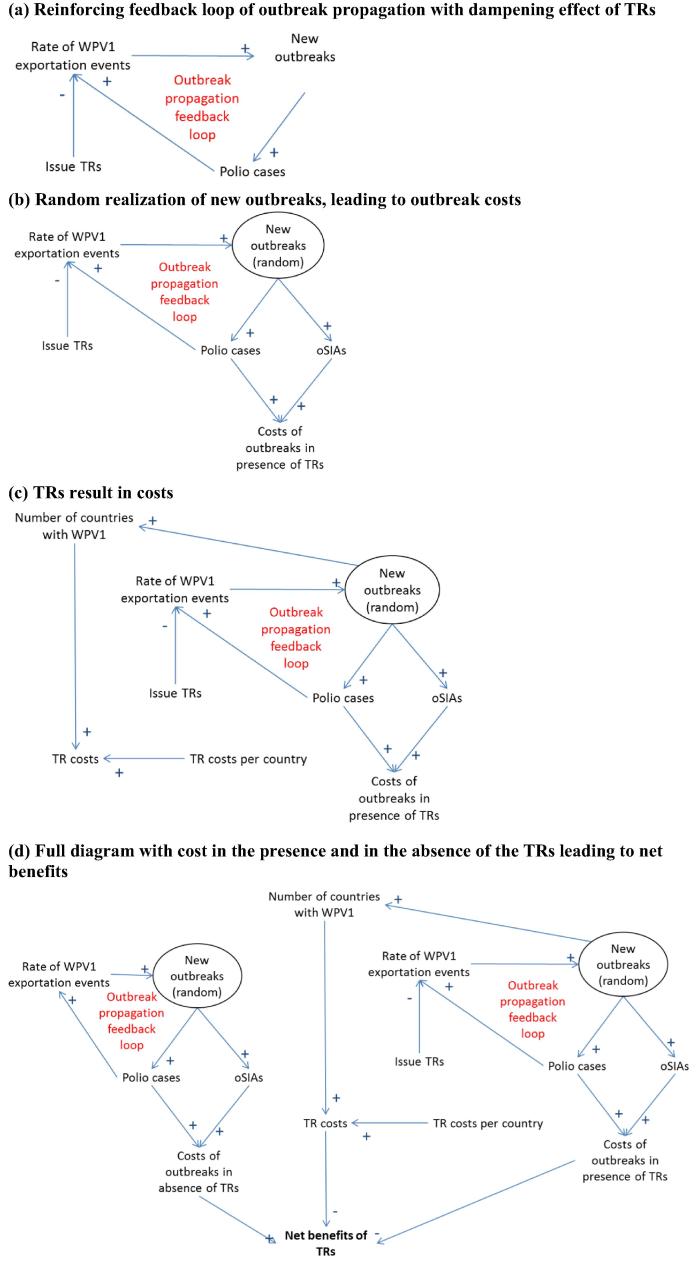
Causal loop diagram illustrating the potential dampening effect of temporary recommendation (TRs) on the reinforcing feedback loop of serotype 1 wild poliovirus (WPV1) outbreak propagation, leading to net health economic benefits. The arrows represent influences and the plus or minus signs show whether all else equal increasing the component at the arrow base increases (plus) or decreases (minus) the component at the arrow tip.

Fig. 1 shows a causal loop diagram of the main components that dynamically interact in the context of TRs (see the Appendix A for a decision tree representation). Fig. 1a shows the fundamental feedback loop that represents the propagation of outbreaks: more new outbreaks lead to more polio cases, which lead to a higher rate of exportation events, which lead to more new outbreaks. Issuing TRs decreases the rate of WPV1 exportation events, which will effectively dampen (i.e., slow down) the outbreak propagation feedback loop. Fig. 1b explicitly characterizes the realization of new outbreaks as random events (depicted using an oval). Each realization implies different numbers of polio cases and outbreak response supplemental immunization activities (oSIAs), which lead to different outbreak costs. Issuing TRs carries costs for each country that needs to implement the TRs, and thus Fig. 1c shows that the occurrence of outbreaks increases the TR costs. Finally, Fig. 1d shows the full diagram with both the costs in the presence of the TRs and the counterfactual outbreak costs in their absence. The difference between these costs represents the incremental net benefits (INBs) of the TRs.

We dynamically and probabilistically account for the relationships depicted in Fig. 1. The model focuses on the effect of TRs on new WPV1 outbreaks in previously WPV1-free countries. Given that no known new WPV1 outbreaks in polio-free countries occurred during 2014–2016 from any of the countries that implemented TRs, the dynamic outbreak propagation model focuses on simulating the occurrence of potential hypothetical outbreaks in the absence of these TRs for the counterfactual scenario. We base these simulations on the average historical rate of 1 WPV1 importation outbreak to polio-free countries per 140 reported WPV1 polio cases during 2004–2013 (i.e., the 10-year time period before the beginning of polio-related TRs) (see Appendix A) [3], [11], [12], [13], [14], [15].

**Fig. 2:**
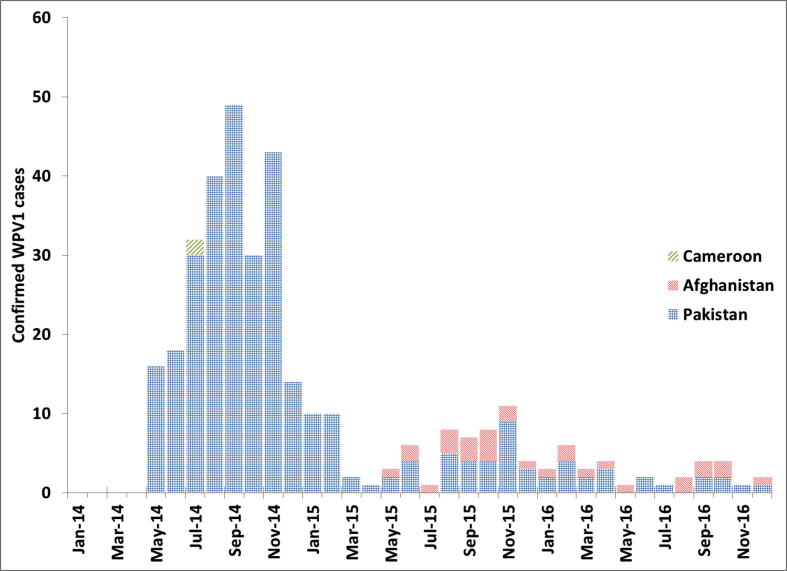
Reported monthly serotype 1 wild poliovirus (WPV1) polio cases from countries with implemented temporary recommendations, 2014–2016.

We assume that the number of WPV1 importation outbreaks in any given month follows a Poisson distribution with a rate equal to the number of reported WPV1 cases in countries that implemented TRs (Fig. 2), multiplied by the average rate of WPV1 importation outbreaks per reported WPV1 case (i.e., 1/140). For every outbreak that occurs, we randomly select an outbreak realization from the 58 outbreaks that occurred during 2004–2013 (see Appendix A). Each outbreak implies a number of oSIA doses used to respond to the outbreak, from which we estimate the vaccination costs of the outbreak, and a list of monthly cases, which we combine with some delay (best estimate 6 months) to characterize the monthly incidence of WPV1 cases that contribute to the probability of generating new outbreaks in future months. We continue until no future cases remain or until reaching the end of the time horizon (i.e., end of 2023), whichever comes first.

Table 1 lists all model inputs and sources, including broad uncertainty bounds for most of the inputs. For each outbreak, we compute the expected direct costs from the number of oSIA doses and the direct treatment costs associated with polio cases using unit costs inputs from prior work [16], [17], [18]. We estimate the TR cost from estimates about the number of travel vaccinations provided to the WHO by countries subject to the TRs, complemented with publicly available national unit costs estimates and estimates from prior publications [16], [17], [18]. We also compute the indirect lifetime costs of lost productivity for each polio case using existing methods that multiply the average number of disability-adjusted life-years per polio case with the income level-specific average annual per-capita gross national income (GNI) [16], [19]. To value the indirect (opportunity) costs of lost productivity associated with time to receive vaccination, we make assumptions about the amount of time spent by travelers to receive vaccine and pro-rate this time cost by the country-specific GNI [20]. In the absence of detail about the age or employment of travelers, we effectively average over all incomes in the country. Finally, to compute the INBs, we subtract the TR costs from the savings associated with prevented outbreaks.

**Table 1:** Model inputs and uncertainty distributions.

Model input [unit]	Assumed parameters of the triangular uncertainty distribution for given model input			Notes
	Mode (i.e., best estimate)	Lower bound	Upper bound	
**Inputs for TR costs estimates:**				
Start of TR implementation [date]				Based on WHO data
-Pakistan	May 2014	–	–	
-Afghanistan	May 2015	–	–	
-Cameroon	May 2014	–	–	

Time under TRs during 2014–2016 [months]				Based on country reports and WHO data
-Pakistan	32	–	–	
-Afghanistan	20	–	–	
-Cameroon	11	–	–	

Per-capita monthly gross national income [$/month]				Based on World Bank data for 2015 [31]
-Pakistan	120	–	–	
-Afghanistan	51	–	–	
-Cameroon	110	–	–	

Vaccinations at POEs, 2014–2016 [people]				Based on country reports
-Pakistan	1,154,513	–	–	
-Afghanistan	301,411	–	–	
-Cameroon	42,507	–	–	

Vaccinations at HFs, 2014–2016 [people]				Based on country reports; uncertainty for Afghanistan reflects discrepancy between sources, with mode assumed equal to the average from both
-Pakistan	13,633,910	**–**	**–**	
-Afghanistan	1,672,721	0	3,345,443	
-Cameroon	0	**–**	**–**	

Average number of POEs over duration of TRs [POEs]				Based on country reports and WHO data
-Pakistan	30	20	40	
-Afghanistan	25	20	30	
-Cameroon	22.5	6	39	

Average salaries for vaccinators at POEs [$/month]				Based on data extracted from cMYPs [21], [32], [33] by WHO
-Pakistan	238	208	267	
-Afghanistan	240	208	267	
-Cameroon	170	142	200	

Administration costs per OPV dose [$/dose]				Based on average routine immunization cost per dose administered, as reported in cMYPs [21], [32], [33]
-Pakistan	1.14	0.5	1.5	
-Afghanistan	0.57	0.3	1.0	
-Cameroon	1.34	0.75	1.75	

Average number of vaccinators per POE [people/POE]	6	2	10	Based on WHO data

Operations cost [%]	10%	0%	25%	Based on WHO data, with upper bound to account for full non-personnel costs

Wastage rate (at HFs or POEs)	0.5	0.3	0.7	Similar to prior estimates [16], [17]

Time spent per vaccination at POE [hours]	0.25	0.1	0.4	Judgment

Time spent per vaccination at HF [hours]	1.0	0.5	2.0	Judgment

OPV price [$/dose]				Similar to prior estimates (converted to year 2015 dollars) [16]
-Low and middle-income	0.12	0.05	0.2	
-High-income	0.16	0.1	2	

**Inputs for outbreak simulation and costs estimates (includes OPV price per dose from above):**				
Average annual gross national income per capita [$/person/year]				Similar to prior estimates (converted to year 2015 dollars) [16]
-Low-income	609	**–**	**–**	
-Lower middle-income	1936	**–**	**–**	
-Upper middle-income	7021	**–**	**–**	
-High-income	38,865	**–**	**–**	

OPV administration costs during SIAs [$/dose]				Use lower middle-income values [16] for upper middle income countries too given types of upper middle-income countries historically affected by outbreaks (e.g., Sudan, Angola)
-Low and middle-income	0.61	0.3	1.0	
-High-income	4.3	2.0	10	

oSIA vs. regular SIA administration costs	1.5	1.0	2.0	Similar to prior estimates [16]

Administered dose per distributed dose	0.5	0.35	0.8	Based on prior wastage corrections [17]

Average treatment cost per polio case [$/case]				Similar to prior estimates (converted to year 2015 dollars) [16]
-Low-income country	660	50	1000	
-Lower middle-income	6600	500	10,000	
-Upper middle-income	66,000	5000	100,000	
-High-income	660,000	50,000	1,000,000	

Outbreak rate [new WPV1 outbreak/reported WPV1 case]	1/140	1/285	1/70	Based on rate during 2004–2013 (see Appendix A)

Delay between WPV1 exportation and first WPV1 importation outbreak polio case [months]	6	1	12	Judgment

Abbreviations: cMYP, comprehensive multi-year plan; HF, health facility; OPV, oral poliovirus vaccine; oSIA, outbreak response SIA; POE, point of entry; SIA, supplemental immunization activity; TR, temporary recommendation; WHO, World Health Organization; WPV1, serotype 1 wild poliovirus

We performed 1000 stochastic iterations of the model with a monthly time step for the outbreak simulation. Each iteration involves both random realizations from all uncertain model inputs and random realizations of outbreaks, which depend on the realized outbreak rate per reported WPV1 case and the delay between exportations and onset of paralysis of the first case.

## 3 Results

**Fig. 3:**
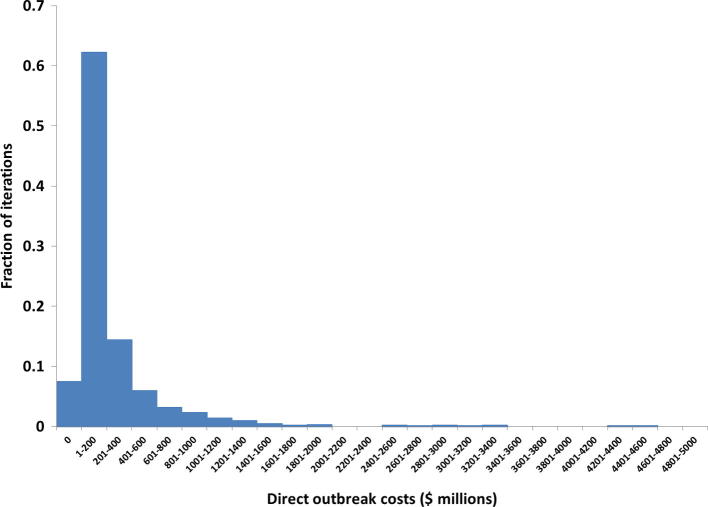
Histogram of direct outbreak-related costs.

With all model inputs at their best estimates (Table 1), the direct costs of implementing the TRs equal almost $24 million, with 87% of these coming from Pakistan (see Appendix A). The indirect costs remain relatively minor at $2.4 million, or 9% of the total direct and indirect costs. These percentages remain similar when fully accounting for model input uncertainty. Fig. 3 shows the distribution of direct outbreak-related costs, which reflect uncertainty in model inputs as well as random variability related to outbreak realizations. If outbreaks directly triggered by the cases in Fig. 2 by chance remain small, as most outbreaks during 2004–2013 (see Appendix A), then with high probability they also end quickly without triggering further outbreaks. However, some WPV1 important outbreaks that occurred during 2004–2013 behaved either explosively or continued for many years, both of which lead to large numbers of WPV1 cases likely to trigger further outbreaks (i.e., they exhibit the outbreak propagation feedback behavior explained in Fig. 1). Of the 1000 model iterations, 75 (7.5%) resulted in no new outbreaks at all, 437 (44%) resulted in 1–4 outbreaks, and 137 (14%) resulted in more than 10 outbreaks. The simulation suggested a very long tail, with a 95th percentile of 18 outbreaks and a maximum of 69 outbreaks through 2023. Outbreaks continued until the end of 2023 in 41 model iterations (4.1%). Fig. 3 shows a very long tail in direct outbreak costs, with a 95th percentile of $960 million and a maximum of $4.5 billion. The largest number of simulated outbreak cases equaled almost 6000.

**Fig. 4:**
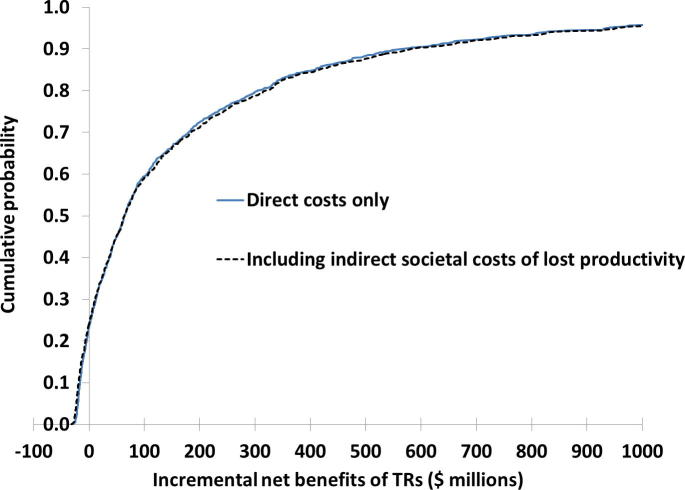
Cumulative distribution function of the incremental net benefits of the temporary recommendations (TRs) issued during 2014–2016.

Table 2 summarizes the expected costs of implementing the TRs during 2014–2016, the expected savings associated with outbreaks prevented, and the expected INBs of the TRs based on all 1000 model iterations. As in Fig. 3, these results account for both the random variability related to outbreak realizations and the uncertainty in the model inputs described in Table 1. Clearly, the expected savings far outweigh the expected costs of the TRs. However, given the very wide uncertainty about the net savings, the lower percentiles of the INBs also include negative values associated with model iterations in which the savings from prevented outbreaks did not exceed the costs of the TRs. Table 2 further shows that inclusion of the indirect costs does not significantly affect the incremental net benefits, in part because indirect costs exist in relation to both implementation of the TRs and the counterfactual outbreaks that occur in the absence of TRs. Fig. 4 provides the full cumulative probability distribution of the INBs from Table 2, showing a 23% chance (24% if we include indirect costs) of negative INBs, but typically much greater positive values for the 77% of model iterations with positive INBs. The INBs of the TRs exceeded $100 million in 41% of model iterations.

**Table 2:** Comparison of expected temporary recommendation (TR) costs and savings from prevented outbreaks and estimated incremental net benefits. Amount in $ million, values in parentheses represent 5th and 95th percentiles, values in square brackets represent the full range.

Result	Direct	Indirect	Total
TR costs	21 (16–27)	2.7 (1.6–4.0)	24 (18–30)
	[12–32]	[1.3–4.7]	[14–34]
Savings from avoided outbreaks	230 (0–960)	8.5 (0–39)	240 (0–980)
	[0–4500]	[0–150]	[0–4600]
Incremental net benefits of TRs	210 (−20 to 940)	5.8 (−3.5 to 37)	215 (−23 to 960)
	[−30 to 4500]	[−4.4 to 150]	[−32 to 4600]

We conducted several univariate sensitivity analyses on the expected INBs (including indirect costs). We observed the greatest impact from changing the basis for outbreak simulations from the last 10 years to 5 years before 2014, during which more outbreaks occurred per reported WPV1 case, but these outbreaks generally remained smaller and shorter in duration (see Appendix A). This change decreased the INBs from $215 to 125 million. Artificially truncating prolonged outbreaks at 5 years instead of 10 years decreased the net benefits to $180 million, while increasing the truncation time to 50 years did not substantially increase the net benefits because outbreaks rarely continued for more than 10 years. Recognizing that our methods potentially counted some planned preventive SIAs (pSIAs) that occurred around the time of outbreaks as oSIAs and that the expected INBs depend approximately linearly on the estimated number of oSIA doses, we found that the expected INBs only become negative if we attribute 94% of assumed oSIA doses to pSIAs doses, which remains very unlikely. A probabilistic sensitivity analysis of the model inputs in Table 1 revealed the relatively weak influence of these model inputs on INBs (because random realizations of outbreaks dominate the uncertainty), with the greatest influence coming from the assumed outbreak rate per reported WPV1 case.

## 4 Discussion

The full characterization of the costs of implementing TRs reveals significant expected direct costs of over $20 million over 3 years (i.e., approximately $7 million per year). For perspective, in 2016, Pakistan reported total annual immunization costs of $235 million [21], with the expected annual TR costs of approximately $7 million representing a small part (3%). In Pakistan, campaigns represent approximately 30% of the total immunization budget (i.e., $75 million per year mainly for polio and measles SIAs), and we should expect costs on this order of magnitude in the event of an imported WPV1 outbreak affecting a similar country. Using actual outbreaks from the last 10 years before the start of the TRs and probabilities based on the rate of WPV1 exportations per reported WPV1 case, we estimate significantly higher expected costs of over $200 million associated with outbreaks prevented by the TRs compared to the costs of the TRs. This high expected value reflects the long tail of possible outbreak-associated costs in the absence of the TRs, with many model iterations leading to more moderate averted outbreak costs. The high risk of outbreaks remains consistent with findings using a differential equation based modeling approach that estimated 665 poliovirus exportations during 2014 alone [7], and with statistical analysis of poliovirus importation outbreaks [8]. As with other polio endgame risks (e.g., containment, immunodeficiency-associated vaccine-derived polioviruses), the challenges come with managing low-probability-high-consequence events [16], [22], [23].

Besides the quantified expected net benefits of the TRs demonstrated in this analysis, the TRs also provide a means to account for the negative externalities that countries that sustain WPV1 transmission impose on other countries by increasing the global risk of WPV1 importation outbreaks. Although countries may not perceive benefits of implementing the TRs within their borders, doing so produces real benefits for other countries. In addition, countries that aggressively implement TRs may also reap some benefits within their borders by effectively reaching seasonal migrant populations that play an important role in sustaining WPV1 transmission (e.g., migrants between Pakistan and Afghanistan).

Although during 2014–2016 no outbreaks occurred in polio-free countries due to virus exported from countries that implemented TRs, we cannot know whether outbreaks would have occurred without implementation of the TRs, thus introducing inherent uncertainty. Similarly, we cannot know whether TRs will continue to prevent them. If WPV1 circulation continues, then it appears likely that eventually exportations may occur in spite of the TRs (i.e., the TRs reduce risks but do not eliminate them). Further, if WPV1 circulation continues in endemic countries and if polio-free countries do not sustain high enough vaccination coverage to protect themselves, then any delay in importations associated with the TRs in endemic countries will imply potentially more explosive outbreaks when the importation occurs (unless the polio-free country already generated a widespread indigenous serotype 1 cVDPV that raised its population immunity to transmission at the expense of cVDPV cases). Thus, despite the expected benefits of TRs, the most important strategy to prevent WPV1 importations remains sustaining high enough population immunity to transmission in all countries.

The TRs differentiate “states currently exporting wild poliovirus or cVDPV,” which must “ensure that all residents and long-term visitors (i.e. >four weeks) of all ages, receive a dose of oral poliovirus vaccine (OPV) or inactivated poliovirus vaccine (IPV) between four weeks and 12 months prior to international travel” from “states infected with wild poliovirus or cVDPVs but not currently exporting,” which should merely “encourage residents and long-term visitors to receive a dose of OPV or IPV four weeks to 12 months prior to international travel [2].” Consequently, the TR costs reported in this study suggests more intense implementation of TRs in countries known to actively export WPV1 compared to those infected with WPV1 but not subject to the same TRs as actively exporting countries. However, increasing the intensity of TRs only after documented WPV1 exportations (and decreasing it in the absence of known WPV1 exportations) may imply that measures only increase after the occurrence of an undesirable event. This reactive nature of the TRs may effectively reduce the ability of the TRs to prevent exportations, which depends on the extent of WPV1 circulation and not on the observation of such an event. Although imposing TRs before evidence of the occurrence of an undesirable event remains politically more difficult, we encourage further discussion about the feasibility of issuing TRs on the basis of extent of WPV1 circulation (e.g., approximated by WPV1 polio incidence) instead of documented WPV1 exportations.

We highlight several limitations with a potential large impact (see Appendix A for a comprehensive list). First, we did not account for the possibility that any WPV1 importations that the TRs prevented would have delayed global WPV eradication. Given estimated costs of over $1 billion per year of delay in global WPV eradication [16], inclusion of potentially averting such costs would substantially increase the expected net benefits of the TRs. We also did not explicitly consider the additional benefits of the TRs of preventing exportations between endemic countries, although this probably represents the most common pathway of WPV1 exportations for Pakistan and Afghanistan. However, given that both countries already carry out intensive SIAs to eradicate WPV1, any cross-border exportations may primarily represent shifts of resources rather than true additional costs. The focus of TRs in Pakistan and Afghanistan on reducing transmission between these two countries may imply a relatively lower effect of WPV1 exportations to other countries, which historically did not experience as many WPV1 importations from Pakistan or Afghanistan as from Nigeria or India [3]. Accounting for the specific geography of countries that implemented TRs rather than on the global average WPV1 importation rate may decrease the expected benefits of the TRs. Finally, by extrapolating from the 2004–2013 experience, the model explicitly assumes the same global population immunity to serotype 1 transmission and outbreak response capacity during 2004–2013 as from 2014 forward. The absence of any recent WPV1 importations may indicate global improvements over time, although it may also reflect the large decrease in global WPV1 incidence and/or effectiveness of the TRs. As the GPEI continues to shift focus on managing OPV cessation, some risk exists that global population immunity will decrease going forward, which would increase outbreak risks and the expected net benefits of the TRs.

The global removal of all serotype 2-containing OPV launched an era of unprecedented low population immunity to serotype 2 transmission [24], [25]. Current experience with cVDPV2 outbreaks in Nigeria and Pakistan represent global emergencies [26]. Decreasing global population immunity to serotype 2 transmission implies a much greater potential for serotype 2 cVDPV (cVDPV2) exportations to cause new outbreaks than in the past and a failure to contain cVDPV2 outbreaks could lead to a need to restart serotype 2-containing OPV in countries currently using OPV [16]. This would imply greater potential benefits of implementing TRs for cVDPV2 outbreaks. However, vaccinating travelers with serotype 2 monovalent OPV increases the risk of reintroducing a serotype 2 live poliovirus into other countries, which can eventually lead to new cVDPV2 outbreaks [27], [28]. Using IPV to implement the TRs would significantly increase the TR costs and would primarily reduce the probability of exporting the cVDPV2 only for individuals with pre-existing immunity from a serotype 2 live poliovirus infection [29], [30]. Thus, estimating the benefits of implementing TRs using IPV for cVDPV2 outbreaks requires further study, because the effectiveness and economics will differ significantly.

## Figures and Tables

**Fig. A1:**
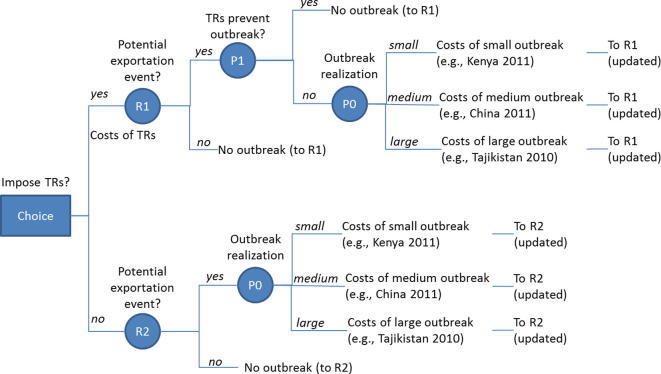
Decision tree for the choice to issue temporary recommendations (TRs) for serotype 1 wild poliovirus (WPV1). Circles with a P indicate one-time probabilities of different outcomes, while circles with R indicate probabilities that apply at each time step and may change with time as new WPV1 outbreaks may occur.

**Fig. A2:**
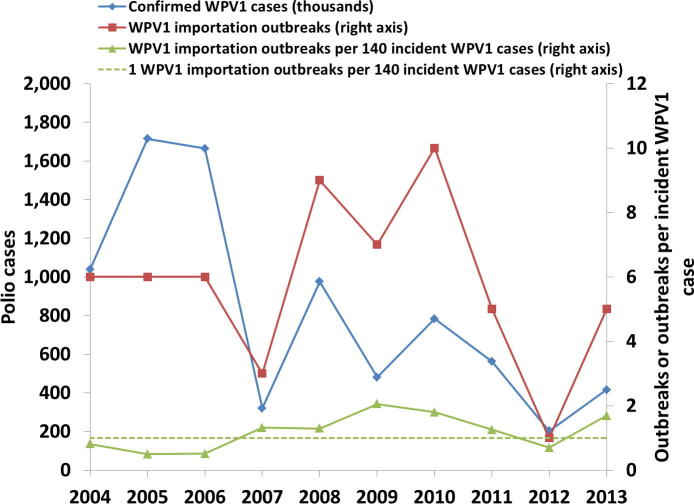
Annual number of confirmed WPV1 cases, WPV1 importation outbreaks into previously polio-free countries, and rate of WPV1 importation outbreaks into previously polio-free countries compared to the average rate over the 10-year time period.

**Table A1:** List of WPV1 importation outbreaks into previously polio-free countries used to estimate the outbreak rate and to draw outbreak realizations.

Index	Name	Income level	First Case	Last case	Cumulative oSIA fraction^a^	# oSIA doses^b^	Reported cases
0	Sudan 2004	UMI	May-04	Jun-09	40.93	309,200,186	222
1	Ethiopia 2004	LOW	Dec-04	Nov-06	8.02	144,687,858	40
2	Botswana 2004	UMI	Feb-04	Feb-04	2.00	480,595	1
3	Mali 2004	LOW	Apr-04	May-05	4.01	16,715,442	19
4	Saudi Arabia 2004	HIGH	Dec-04	Dec-04	1.45	6,491,326	1
5	Guinea 2004	LOW	Jun-04	Dec-04	6.00	13,523,555	7
6	Yemen 2005	LMI	Feb-05	Feb-06	10.39	51,939,440	479
7	Somalia 2005	LOW	Jul-05	Mar-07	24.84	42,732,544	228
8	Indonesia 2005	LMI	Mar-05	Feb-06	6.29	156,248,793	305
9	Eritrea 2005	LOW	Apr-05	Apr-05	3.00	1,981,080	1
10	Angola 2005	UMI	Apr-05	Nov-06	9.63	60,338,787	11
11	Nepal 2005	LOW	Aug-05	Oct-05	1.18	5,475,608	4
12	DRC 2006	LOW	Feb-06	Dec-11	19.26	317,816,435	250
13	Nepal 2006	LOW	Mar-06	Dec-06	4.48	23,886,182	5
14	Kenya 2006	LOW	Sep-06	Nov-06	0.76	6,264,637	2
15	Namibia 2006	UMI	May-06	Jun-06	3.00	5,639,533	19
16	Bangladesh 2006	LOW	Jan-06	Nov-06	9.23	259,147,374	18
17	Niger 2006	LOW	Apr-06	Oct-06	4.28	18,602,016	7
18	Myanmar 2007	LOW	Mar-07	May-07	3.91	30,137,358	11
19	Angola 2007	UMI	Apr-07	Jul-11	26.01	167,936,984	80
20	Niger 2007	LOW	Mar-07	Oct-07	3.22	14,804,647	10
21	Benin 2008	LOW	Apr-08	Apr-09	6.54	21,942,131	25
22	Burkina Faso 2008	LOW	Jun-08	Oct-09	11.69	65,261,216	21
23	Ghana 2008	LOW	Sep-08	Nov-08	4.88	30,555,029	8
24	Ethiopia 2008	LOW	Mar-08	Apr-08	0.99	14,242,520	3
25	CAR 2008	LOW	Apr-08	Dec-08	8.00	7,234,104	3
26	Cote d'Ivoire 2008	LMI	Dec-08	Aug-09	9.00	66,488,292	27
27	Mali 2008	LOW	Aug-08	Nov-09	7.81	41,143,684	3
28	Niger 2008	LOW	Jan-08	May-09	12.43	57,727,368	9
29	Togo 2008	LOW	Oct-08	Mar-09	4.00	7,329,156	10
30	Kenya 2009	LOW	Feb-09	Jul-09	1.23	10,854,032	19
31	Burundi 2009	LOW	Sep-09	Sep-09	2.00	3,770,103	2
32	Sierra Leone 2009	LOW	Jul-09	Feb-10	8.88	9,601,800	12
33	Mauritania 2009	LMI	Oct-09	Apr-10	9.90	8,055,006	18
34	Liberia 2009	LOW	Apr-09	Sep-10	15.59	16,083,964	13
35	Guinea 2009	LOW	Apr-09	Nov-09	10.99	31,644,182	40
36	Uganda 2009	LOW	Jan-09	May-09	2.28	17,557,598	8
37	Uganda 2010	LOW	Sep-10	Nov-10	1.94	14,688,757	4
38	Liberia 2010	LOW	Mar-10	Sep-10	12.00	10,600,894	2
39	Mali 2010	LOW	Mar-10	May-10	6.29	37,079,051	3
40	Senegal 2010	LMI	Jan-10	Apr-10	7.19	19,538,311	18
41	Nepal 2010	LOW	Feb-10	Aug-10	6.15	34,871,211	6
42	Tajikistan 2010	LOW	Feb-10	Jul-10	6.30	15,415,095	460
43	Kazakhstan 2010	LMI	Aug-10	Aug-10	1.46	3,952,878	1
44	Turkmenistan 2010	UMI	Jun-10	Jun-10	3.77	4,613,556	3
45	Russian Federation 2010	HIGH	May-10	Sep-10	0.22	4,452,800	14
46	Republic of Congo 2010	LMI	Sep-10	Jan-11	7.18	29,094,218	455
47	China 2011	UMI	Jul-11	Oct-11	0.51	43,700,000	21
48	Niger 2011	LOW	Jul-11	Dec-11	7.95	41,639,025	4
49	CAR 2011	LOW	Sep-11	Dec-11	7.76	7,267,770	4
50	Kenya 2011	LOW	Jul-11	Jul-11	2.02	17,461,569	1
51	Gabon 2011	UMI	Jan-11	Jan-11	3.00	5,554,170	1
52	Niger 2012	LOW	Nov-12	Nov-12	6.17	34,783,063	1
53	Somalia 2013	LOW	Apr-13	Aug-14	21.92	70,840,604	199
54	Syria 2013	LMI	Jul-13	Jan-14	15.20	48,637,546	36
55	Ethiopia 2013	LOW	Jul-13	Jan-14	4.68	61,139,110	10
56	Kenya 2013	LOW	Apr-13	Jul-13	6.16	48,992,904	14
57	Cameroon 2013	LMI	Oct-13	Jul-14	16.35	79,329,984	9

HIGH, high-income country; LMI, lower middle-income country; LOW, low-income country; UMI, upper middle-income country.

a Based on sum of fraction of country targeted for all SIAs between the time of the first case and 12 months after the time of the last case or onset of the first case of a new WPV1 importation outbreak in the same country; in the event of simultaneous SIAs targeting more than 100% of the country, we use include only the SIA designed as “parent” in the SIA planning tool.

b Based on required doses by SIA planning tool.

**Table A2:** Breakdown of undiscounted TR costs assuming best estimates from Table 1 for all model inputs.

Model output related to TR costs	Pakistan	Afghanistan	Cameroon	All 3 countries
Average monthly vaccinations				
-POEs	36,000	9400	3900	55,000
-HFs	430,000	52,000	0	580,000

Cumulative vaccine costs (incl. wastage)				
-POEs	280,000	72,000	10,000	350,000
-HFs	3,300,000	400,000	0	3,700,000

Cumulative administration costs (incl. operations)				
-POEs	1,500,000	1,300,000	280,000	2,800,000
-HFs	16,000,000	950,000	0	17,000,000

Total direct costs	21,000,000	2,700,000	290,000	24,000,000

Cumulative person-months of time to receive vaccines				
-POEs	400	105	15	520
-HFs	71,000	3700	0	75,000

Total indirect costs	2,300,000	120,000	1600	2,400,000

**Table A3:** Rank correlations between model inputs in Table 1 and the INBs of the TRs (including indirect societal costs of lost productivity), sorted from high to low absolute values.

Model input (see Table 1)	Rank correlation with the INBs
Outbreak rate	0.29
Administered dose per distributed dose	0.26
OPV administration costs during SIAs	0.083
OPV price	0.058
Operations costs	−0.048
Wastage rate (at HF or POE)	0.047
Average number of POEs over duration of TRs	−0.042
Average number of DALYs associated with a paralytic polio case	−0.036
oSIA vs. regular SIA administration costs	0.031
Time spent per vaccination at POE	0.031
Average monthly salary for vaccinators	0.029
Treatment cost	0.021
Delay between WPV1 exportation and first WPV1 importation outbreak polio case	0.018
Vaccinations at HFs, 2014–2016	0.013
Time spent per vaccination at HF	−0.011
Average number of vaccinators per POE	−0.0011
Administration costs per OPV dose	−0.0010

**Table A4:** Table of model limitations and their possible effect on the expected net benefits of the TRs.

Limitation	Effect if included	Potential impact on expected INBs of TRs
**Limitations related to scope:**		
Costs of possible delay in WPV1 eradication caused by WPV1 importation outbreaks not included	Would increase benefits of TRs	Large
Effect of TRs in importations in endemic countries not included	Would increase benefits of TRs	Medium
Possible future IPV use for oSIAs not considered	Would increase benefits of TRs	Medium
Prevented outbreaks beyond 2024 not included	Would increase benefits of TRs	Small
Effect of TRs on population immunity in countries implementing TRs excluded	Would increase benefits of TRs	Small
Returning refugee vaccination and cross-border SIAs reported by Afghanistan and Pakistan as part of TR activities not included	Higher TR costs but also higher benefits	Small
Significant impact of one known outbreak of asymptomatic WPV1 transmission not considered (i.e., Israel 2013)	Would increase benefits of TRs	Small
Incremental cost of 7000 vaccinations with IPV instead of OPV in Cameroon excluded	Would decrease benefits of TRs	Small

**Technical limitations:**		
Specific countries most at risk from WPV1 importations from Pakistan (and Afghanistan and Cameroon) not explicitly considered	Would probably decrease benefits of TRs because Pakistan historically did not cause large outbreaks	Large
Extrapolation from 2004–13 experience to 2014–2016 does not account for changes in population immunity to transmission or outbreak response ability	Both directions possible, but likely would decrease benefits of TRs	Large
SIAs that would have been conducted regardless of outbreak occurrence not removed from historic outbreak list (Table 1)	Would decrease benefits of TRs	Medium
Multiple historic WPV1 importation events during same year counted as a single outbreak	Both directions possible, as inclusion would increase rate of outbreaks but also increase probability of small outbreaks	Small
Cost of all oSIA discounted towards year of first case, even if they continue for multiple years	Would decrease benefits of TRs	Small
Same historic outbreak may randomly get selected multiple times	Both directions possible	Small

## Appendix A

Fig. A1 provides a decision tree that explicitly shows the choice and the potential effects of issuing TRs on the random occurrence of outbreaks. In Fig. A1, potential exportation events may occur at some rate regardless of the choice to implement TRs. We assume that the rate depends on global WPV1 prevalence, for which the incidence of reported WPV1 cases provides an indicator. Implementation of the TRs results in some probability that a potential exportation event (i.e., one that would result in a WPV1 outbreak in the absence of the TRs) does not lead to an outbreak (i.e., denoted by node P1). Therefore, the number of cases differs in the presence of the TRs and in the counterfactual scenario without the TRs, which leads to a difference in the future rate of outbreaks between the top part of tree (i.e., rate denoted by the node R1) and the bottom part of the tree (i.e., rate denoted by the node R2). If an outbreak occurs, then a probability distribution (i.e., denoted by the node P0) determines the actual outbreak realization. To illustrate the concept, Fig. A1 shows three possible types of outbreaks, which represent conceptual categories into which the numerous possible modeled outbreaks may occur (i.e., in the actual model, a branch exists for each of the many possible outbreaks). Once an outbreak occurs, then this implies a need to update the future outbreak rate because the outbreak increases the number of WPV cases (see Fig. 1a), as indicated to the right of each outbreak realization branch in Fig. A1. In the real world, the location and size of an outbreak depends on numerous factors, including proximity to exporting country, population immunity to transmission in importing country, and outbreak response. However, for simplicity, we assume the same probability distribution P0 in the presence of TRs and for the counterfactual scenario. If the expected costs of the TRs for all outbreaks that occur with the TRs remain smaller than the expected costs of all the outbreaks that would occur for the counterfactual scenario, then implementation of the TRs represents an economically preferable option.

Fig. A2 shows the annual number of WPV1 importation outbreaks juxtaposed to the reported annual number of confirmed WPV1 cases. Over the 10-year time span, on average approximately one importation outbreak occurred for every 140 reported WPV1 cases. The ratio for individual years varied somewhat around this average, as shown by the green curves in Fig. A2.

Table A1 lists all 58 WPV1 importation outbreaks during 2004–2013, with attributes of the outbreak country and epidemiology based on Global Polio Eradication Initiative data and the published literature [3], [11], [12], [13], [14], [15]. The table lists only the total number of cases associated with the importation outbreak, but in the actual model we use monthly case numbers, which contributes to the rate of importation outbreaks going forward.

Table A2 shows the estimates of the TR implementation costs for the three countries that reported significant efforts to implement the TRs, based on best estimates from Table 2, to illustrate the typical breakdown. Table A2 clearly shows the importance of including administration costs at the HFs, which represent almost 70% of the total direct costs. Inclusion of significant vaccine wastage (Table 1) further increases the best estimates of total costs. Pakistan emerges as the main driver of all cost components, representing 88% of all direct and indirect costs. The indirect costs remain relatively minor at 9% of the total direct and indirect costs. Fully accounting for model input uncertainty produces a similar pattern of major cost drivers, with some changes in the precise ratios due to the use of asymmetric distributions for some inputs in Table 1. Overall, vaccinations at HFs accounted for 86% of average direct costs (including both vaccines and administration), Pakistan accounted for 88% of the average direct and indirect costs, and indirect cost accounted for 11% of the average direct and indirect costs.

Table A3 lists the results of a probabilistic sensitivity analysis that explores the influence of the model inputs in Table 1 on the INBs (including indirect societal costs of lost productivity). We measure importance using the (Spearman) rank correlation, with values closer to one indicating a strong increasing (not necessarily linear) relationship between the model input and the INBs, values closer to negative one indicating a strong decreasing relationship between the model input and the INBs, and values near zero suggesting little influence of the model input on the INBs [34]. Table A3 suggests, not surprisingly, that the assumed outbreak rate per reported WPV1 case represents the most important model input. The number of administered doses per distributed dose during oSIAs represents the next most important model input. We multiply the estimated required oSIA doses in Table A1 by this number to estimate the administration costs during all oSIAs, and the range from 0.3 to 0.8 thus spans a more than twofold increase in the oSIA costs, which account for the largest component of the INBs (Table 2). All other model inputs remain much less influential. Due to the importance of random variability in outbreak realizations, the relative contribution of all model inputs to the uncertainty in the INBs remains relatively modest (i.e., rank correlations below 0.3).

In addition to the uncertainty and sensitivity, Table A4 lists more structural limitations of the analysis and their likely effect on the expected benefits of the TRs.
